# *Mycobacterium intracellulare subsp. chimaera* from Cardio Surgery Heating-Cooling Units and from Clinical Samples in Israel Are Genetically Unrelated

**DOI:** 10.3390/pathogens10111392

**Published:** 2021-10-27

**Authors:** Mor Rubinstein, Rona Grossman, Israel Nissan, Mitchell J. Schwaber, Yehuda Carmeli, Hasia Kaidar-Shwartz, Zeev Dveyrin, Efrat Rorman

**Affiliations:** 1 National Public Health Laboratory, Ministry of Health, Tel Aviv-Yafo 6810416, Israel; Israel.nissan@moh.gov.il (I.N.); hasia.shwartz@PHLTA.HEALTH.GOV.IL (H.K.-S.); zeev.dveyrin@PHLTA.HEALTH.GOV.IL (Z.D.); efrat.rorman@PHLTA.HEALTH.GOV.IL (E.R.); 2 Government Central Laboratories, Ministry of Health, Jerusalem 9134302, Israel; rona.grossman@moh.gov.il; 3 National Center for Infection Control, Ministry of Health, Tel Aviv-Yafo 6423906, Israel; mitchells@tlvmc.gov.il (M.J.S.); yehudac@tlvmc.gov.il (Y.C.); 4 Sackler Faculty of Medicine, Tel Aviv University, Tel Aviv-Yafo 6997801, Israel; 5 National *Mycobacterium* Reference Centre, Tel Aviv-Yafo 6810416, Israel

**Keywords:** *Mycobacterium chimaera*, NTM, metagenomic binning

## Abstract

Non-tuberculous mycobacteria (NTM) are opportunistic pathogens that cause illness primarily in the elderly, in the immunocompromised or in patients with underlying lung disease. Since 2013, a global outbreak of NTM infection related to heater-cooler units (HCU) used in cardio-thoracic surgery has been identified. This outbreak was caused by a single strain of *Mycobacterium intracellulare subsp. chimaera*. In order to estimate the prevalence of this outbreak strain in Israel, we sampled *Mycobacterium intracellulare subsp. chimaera* from several HCU machines in Israel, as well as from patients, sequenced their genomes and compared them to the outbreak strain. The presence of mixed mycobacteria species in the samples complicated the analysis of obtained sequences. By applying a metagenomic binning strategy, we were able to obtain, and characterize, genomes of single strains from the mixed samples. *Mycobacterium intracellulare subsp. chimaera* strains were compared to each other and to previously reported genomes from other countries. The strain causing the outbreak related to the HCU machines was identified in several such machines in Israel but not in any clinical sample.

## 1. Introduction

Non-tuberculous mycobacteria (NTM) are ubiquitous environmental bacteria found primarily in soil and water. They are considered opportunistic pathogens; however, the occurrences of disease and even death caused by these bacteria in recent years are increasing [1,2,3,4,5]. These bacteria are resistant to many drugs and disinfectants [3,6,7]. *Mycobacterium intracellulare subsp. chimaera (M. chimaera*, previously classified as the separate species *Mycobacterium chimaera*) [8] is a slow-growing NTM, which is now considered a subspecies of *Mycobacterium intracellulare*, with high similarity to the subspecies *intracellulare* [8,9].

In the second decade of the 21 century, a global outbreak of *M. chimaera* disseminated infection and endocarditis occurred among patients who had undergone cardiopulmonary bypass surgeries [10,11,12,13]. The source of infection was identified as contaminated water in heater-cooler units (HCU), which regulate the temperature of blood and cardioplegia solution during open-heart surgery. Aerosols from the HCU, containing the pathogenic bacteria, spread in the operating room, infecting the patients’ open chest or grafts [14]. *M. chimaera* detected at the manufacturing site of the LivaNova (formerly Sorin) HCU suggested this was the source of the contamination [15].

In order to identify the source of the outbreak, the European Union launched an epidemiological investigation, in which genomes of 250 isolates, from patients, HCUs and the water supply in five different countries were sequenced, and the results were published in 2017 by Van Ingen et al. [16]. A phylogenetic analysis of the genomes divided most of the strains into two main groups. Almost all the strains obtained from patients who had undergone cardiopulmonary bypass surgery, belonged to a closely related subgroup which the authors designated subgroup 1.1. Van Ingen et al. also identified specific SNP signatures for some of the phylogenetic groups, including the outbreak subgroup 1.1 [16]. Other studies supported the finding that a single strain of *M. chimaera* was involved in this outbreak [17,18,19].

In addition to the clinical strains from patients related to the outbreak, the outbreak subgroup 1.1 included most of the strains from HCUs manufactured by LivaNova as well as samples taken from the LivaNova production site. Interestingly, other strains of *M. chimaera* were detected in water taken from HCUs manufactured by another company, Maquet, but these strains were genetically different from the strain causing the outbreak [16]. The mechanical characteristics of the LivaNova/Sorin 3T model of HCUs favored the spread of bacteria containing aerosols [14]. Following these findings, LivaNova modified this HCU device to improve its safety.

The Israel Ministry of Health (MOH) was notified in November 2016 by LivaNova of the NTM infections associated with use of its Stöckert 3T heater-cooler devices. At the time, 25 such devices were distributed among 11 hospitals throughout Israel performing open-heart surgery. The following steps were undertaken:

1. General hospital CEOs were notified, and asked to inform relevant staff members of the outbreak and nature of invasive NTM infections associated with use of these machines;

2. The directors of the clinical microbiology laboratories in general hospitals were asked to retroactively relay information on any pertinent cultures from patients who had undergone cardiac surgery with use of a heater-cooler device;

3. The public was notified via press release and a media interview of the outbreak and those having undergone open-heart surgery since 2011 were asked to report any suspicious symptoms or signs to their medical providers for evaluation;

4. CEOs of hospitals with the heater-cooler devices in use were asked to ensure that updated safety instructions issued by the manufacturer, and additional measures requested by the MOH, were strictly followed;

5. Hospitals using the machines were required to submit water samples for NTM, document device maintenance, and report bimonthly bacterial colony counts from water in the devices.

6. The manufacturer, in conjunction with the Ministry of Health, embarked a staggered recall of the devices to their plant in Europe, where they underwent cleaning, as well as installation of safety features that would render patients no longer vulnerable to infection due to contaminated exhaust from the water. This process was completed by mid-2018.

In order to estimate the infection risk, water samples from HCUs at 10 medical centers were sent to the Israeli public health laboratories for total bacterial count and specific identification of *Mycobacterium* contamination.

Fortunately, no clinical infections with *M. chimaera* related to use of the HCU were reported in Israel. Nevertheless, to characterize the epidemiology of *M*. *chimaera* in Israel and to evaluate the possibility of HCU-related *M. chimaera* infections, we sequenced the genomes of *M*. *chimaera* from the HCUs, as well as all the clinical *Mycobacterium* isolates in the Israeli mycobacterial reference laboratory that were identified as *M. chimaera.*

We found that some of the HCU cultures contained mixed mycobacterial species, which complicated the identification of *M. chimaera* strains. Environmental samples are often comprised of mixed mycobacteria strains. Isolating each NTM type is difficult, which makes deep sequencing analysis challenging. Several approaches have been applied to the task of deciphering bacterial strains from whole genome sequencing of a mixed culture. Eyre et al. [20] used a maximum likelihood-based model to identify two different strains of *Clostridium difficile* in short read WGS from mixed infection samples. However, their method relies on a previously constructed panel of known haplotypes that ideally include the strains. The QuantTB method [21] that detects mixed infection tuberculosis also uses a reference dataset of MTB genomes. Yang et al. introduced a tool for identifying strains of *Salmonella enterica* in samples from mixed infections, which also uses a database of known strains [22]. DESMAN [23] uses metagenomic binning and core genes to identify strains in mixed genome samples without a strain database. Metagenomic binning is a process that clusters environmental shotgun reads or their assembled contigs back into the taxa composing the sample. It is usually applied to reconstruct nearly complete genomes from metagenomic samples containing a large number of microorganisms. To address the challenge of mixed samples we formulated an in-house method, which involved de novo assembly and metagenomic binning followed by SNP identification and genotyping, by comparing our draft genomes to both a reference genome and a SNP-containing reference genome. Herein, we report the results of this analysis.

## 2. Results

### 2.1. Whole Genome Sequencing and Species Identification

We included in our study all eight *M. chimaera* isolates obtained from clinical samples during 2017 in Israel. These isolates were all obtained from sputum taken from patients suffering from chimaera pulmonary disease. In addition, we included one previous *M. chimaera* isolate obtained from chest biopsy in 2014 that was initially designated as *M. intracellulare*, and one *M. intracellulare* sample isolated from a patient’s pleural fluid in 2013. Besides the clinical samples, we also included thirteen samples of HCU water from ten medical centers that were cultured in the Israeli Mycobacterial Reference Laboratory, and identified as *Mycobacterium intracellulare subsp. chimaera*. All the sampled HCU devices were of the 3T model manufactured by LivaNova/Sorin, the same model that was linked to the global outbreak of *M. chimaera* following open-heart surgery (Table 1).

For initial identification, we used a molecular kit that is based on PCR and proprietary probes (GenoType NTM-DR assay, Hain Lifescience, Nehren, Germany). Two of the HCU samples were identified as mixed samples containing *M. chimaera* (Table 1).

Short reads whole genome sequencing (WGS) of these samples were obtained. We further identified the bacterial species in each sample by assigning taxa to the short sequence reads. The most abundant annotation in all samples was the genus *Mycobacterium* (53–83% of the reads, data not shown). On the species level, in clinical sample M10, originally identified as *M. intracellulare* by Hain Lifescience GenoType assay, the most abundant species-level annotation was indeed *M. intracellulare*. In two other clinical samples, M8 and M9, the most abundant species-level annotations were *Shewanella decolorationis* and *Bacillus azotoformans*, respectively, and *M. chimaera* was only the second most abundant species. However, these samples had 60% and 58% of their reads annotated as *Mycobacterium*, respectively. Therefore, it is safe to conclude that *M. chimaera* was the most abundant species in these samples as well. In seven of the samples from HCU devices, the most abundant species-level annotation was *Mycobacterium gordonae,* another NTM species (Table 2).

Since some of our samples were not pure *M. chimaera*, it was impossible to use the short read sequence for identifying the outbreak *M. chimaera* strain in a straightforward manner. We, therefore, applied the following steps:

Short reads in each sample were assembled de novo into contigs.

Contigs were binned into bacterial species, using metagenomic binning. Each sample resulted in one or two bins (Table 2 and Table S1). Each bin can be seen as an equivalent to genome sequencing of an isolate.

The species of each bin was identified by finding the genome most similar to it, from the collection of all publicly available genomes (Table 2).

A single bin was retrieved in each of our clinical samples, corresponding to one species. Most of these bins were more similar to *M. chimaera* genomes than any other genome, except for two cases. The bin produced from sample M10 was, as expected, most similar to a *M. intracellulare* genome, and sample M2, which was surprisingly most similar to a Mycobacterium sp. TKK-01-0059 genome, a poorly characterized species of the *Mycobacterium tuberculosis* complex (Table 2). However, this bin also showed high similarity to a genome of *M. yongonense,* a sub species of *M. intracellulare* (839/1000 k-mers in MASH analysis [24]), and lower similarity to a genome of *M. chimaera* (588/1000 k-mers). It cannot be ruled out that sample M2 is a strain of *M. chimaera*, which is distant from the strains with publicly available genomes.

The situation was different among samples taken from HCU devices. Samples M16-M19 and M21 had two metagenomic bins in each of them, one corresponding to *M. chimaera* and one to *M. gordonae*. The metagenomic binning process resulted in a single assembly bin, identified as *M. gordonae* by comparison to publicly available genomes, in samples M22 and M23. While a more sensitive PCR-based method (Hain Lifescience GenoType NTM-DR assay) was able to identify two different *Mycobacterium* species in samples M22 and M23, it is possible that the amount of M. chimaera in these samples was too low to be detected in the NGS sequencing by metagenomic sequencing. M19, M21, M22 and M23 had a relatively high proportion of reads annotated as *M. gordonae* (Table 2 column 5). Sample M20, also taken from a HCU, was divided into one bin of *M. chimaera* and one bin most similar to the genome of a *Mycobacterium* species isolated from a drinking water system in Illinois, USA [25]. HCU samples M11–M15 all had a single bin corresponding to *M. chimaera*.

For the rest of our study, we used only the assembly bins identified as *Mycobacerium intracellulare*, including the assembly bin from sample M2. The total length of these assemblies ranged between 5.3 Mbp and 6.9 Mbp, and the GC content between 67.3% and 68.1% (Table S1). In comparison, the published complete genome length of the *M. chimaera* reference strain DSM-44623 and strains Zuerich-1 and Zuerich-2 are 6.1 Mbp, 6.4 Mbp and 6.5 Mbp respectively, and their GC content are 67.7%, 67.5% and 67.4%, respectively.

### 2.2. Identification of Known Phylogenetic Groups by Specific SNP Signatures

**Figure 1:**
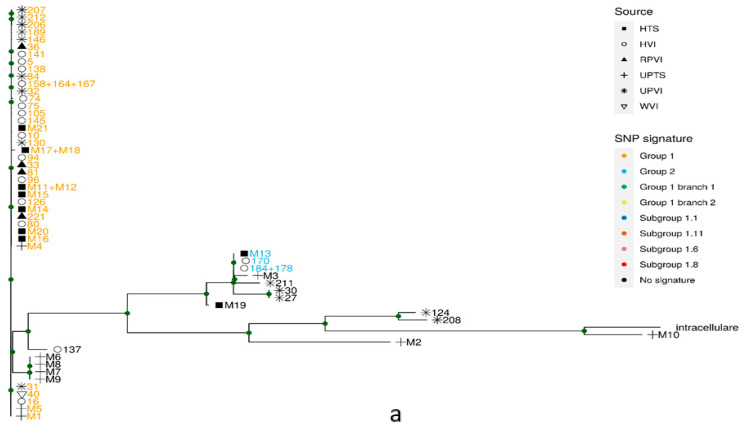

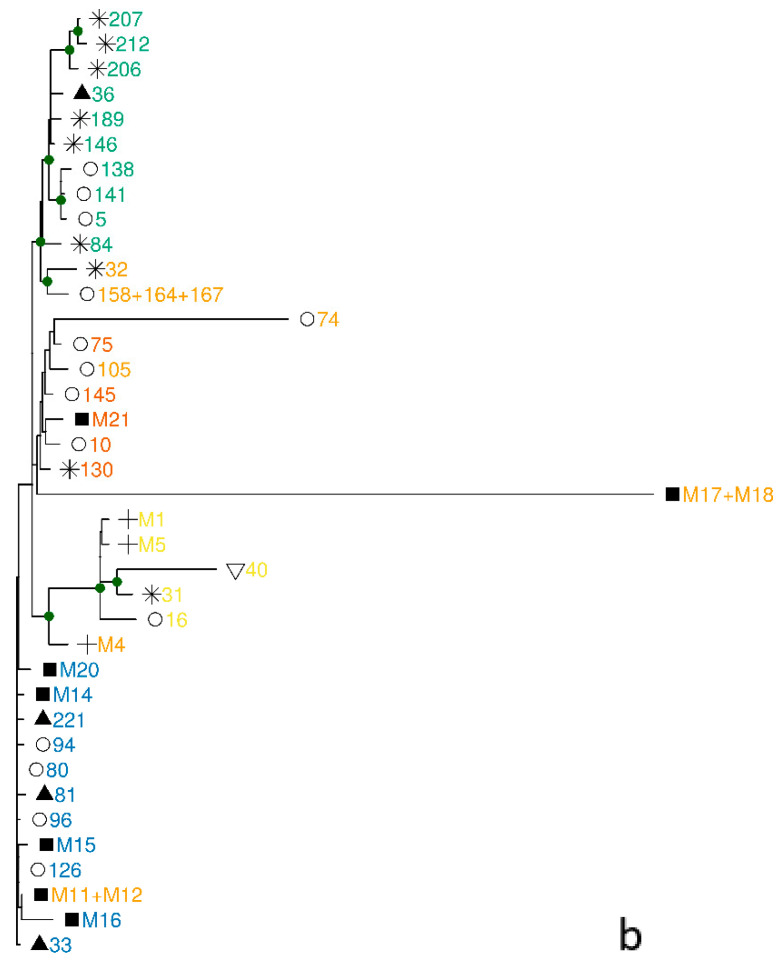
Phylogeny. Maximum likelihood phylogenetic trees based on SNPs identified in the bin assemblies of isolates from this study (Table 1) and selected isolates from the EU study published in 2017 in Lancet Infect Dis [16]. The shapes of the tips represent the isolate source. HTS: HCU device, this study; HVI: HCU device, van Ingen et al.; RPVI: related patient (a patient which has undergone a cardio thoracic surgery), van Ingen et al.; UPVI: unrelated patient, van Ingen et al.; UPTS: unrelated patient, this study; WVI: water dispenser, van Ingen et al. Sample names are colored by specific SNP signatures. Nodes supported by more than 90% bootstraps are marked in green dots. (**a**) All samples; (**b**) samples assigned to group 1 by its specific SNP signature.

We searched our assembled genomes for the SNP signatures defined by van Ingen et al. [16]. Twelve of the samples had a SNP signature assigning them to phylogenetic group 1, the largest phylogenetic group in that study, which included 200 isolates (Figure 1a). Within these 12 samples, two samples were further assigned to branch 2, one to subgroup 1.8, and six to subgroup 1.1, a tightly related phylogenetic subgroup related to the outbreak, by this subgroup’s SNP signature (Figure 1b). Three samples were assigned to group 1 but not to any of its subgroups or branches. In addition, one of our isolates was assigned to van Ingen et al.’s group 2 and, within it, to subgroup 2.1, by their specific SNP signatures (Figure 1a).

The remaining eight genomes were not assigned to any of the groups defined by specific SNP signature. Note that not every phylogenetic sub-type of *M. chimaera* has a SNP signature that can be used for its identification [9].

All isolates in our study that were assigned to subgroup 1.1, the outbreak subgroup, originated from HCU devices, which is in accordance with the fact that no clinical sample was taken from patients who had undergone cardio-thoracic surgery in the past.

### 2.3. Phylogeny

We identified SNP loci, relative to the *M. chimaera* reference strain DSM-44623, in the assembled bin of our isolates, as well as in some isolates analyzed by van Ingen et al. [16] (see next section), and *M. intracellulare* strain MOTT-2. These SNPs were used for building a maximum likelihood phylogenetic tree (Figure 1a). The phylogenetic tree branching pattern agreed with the assignment of isolates by van Ingen et al.’s group-specific SNP signatures [16].

Our samples have a wide genetic variability (Figure 1a). Sample M10, identified as *M. intracellulare* in all previous analyses, is most similar to other *M. intracellulare* genomes. Perhaps surprisingly, the same is true for sample M2 obtained from clinical sputum. Four of our samples, M6–M9, were obtained from clinical samples clustered in one similar, yet not identical, group. The largest group of our samples, comprised of 12 samples, clustered together with isolates assigned by van Ingen et al. [16] to group 1.

To explore the diversity within group 1, a separate maximum likelihood phylogenetic tree was built based on these isolates (Figure 1b). This tree too revealed a close agreement with SNP signatures. Most importantly, six isolates from HCUs clustered together with isolates from the outbreak subgroup described by van Ingen et al. [16]. These isolates also belong to this subgroup based on their signature SNPs.

### 2.4. Validation of the Results

To validate our procedures, we downloaded the raw genomic sequences of some isolates analyzed by van Ingen et al. [16], representing the full genetic variability in that study, and applied the same methods used on our isolates, including taxon annotation of short reads (not shown), de novo assembly followed by metagenomic binning and SNP identification. We used group-specific SNP signatures to assign these genomes back to their phylogenetic groups, and the original groups and subgroups were retrieved. Isolates that were part of the same branch in Ingen et al.’s phylogenetic analyses, exhibited similar clustering when analyzed with this study pipeline and used in phylogenetic trees (Figure 1).

We performed an in silico simulation, creating mixtures of short reads from *M. chimaera* and *M. gordonae* and applied the same method used here to identify SNPs, produce genotype calls and construct a phylogenetic tree, in order to explore the capacity of our method to identify strains from mixed samples. The Appendix A gives a detailed report of this analysis. In short, we were able to identify the original strains from the mixtures, and differentiate between different strains (Figures S1 and S2).

Ten of our samples, M1-10, were obtained from clinical samples, and can be assumed to belong to a single clone of Mycobacterium. To validate our results further, we mapped the sequence reads from theses samples to the *M. chimaera* reference genome and looked for SNPs. Among the SNPs identified in both methods, the base call of our clinical samples is identical in 95–100% of the cases, depending on the strain (average of 99%). The subtype signature SNPs were identical to those obtained with metagenomic binning (data not shown).

## 3. Discussion

The aim of this study was to characterize the *M. chimaera* strains in Israel, in both HCUs and clinical samples, and find out whether the global outbreak strain is present among them.

The mixed nature of samples in this study made it difficult to characterize the strains comprising them, and led us to use an un-orthodox path of bioinformatics analysis. The relative similarity between *Mycobacterium* genomes made the use of mapping to a reference genome inappropriate, since reads from one species of bacterium in the sample could be cross-mapped to a genome of a different bacterium. In addition, the mix of intra-species and inter-species variation may interfere with the identification of strains. In contrast, the use of assembled contigs, which are much longer, enabled the separation of sequences into bins representing the different bacteria. The metagenomic binning approach served as an in silico isolation method. The agreement in genome size and GC content of the bins we annotated as *M. chimaera*, with those characteristics of known genomes of *M. chimaera*, supports this annotation. Moreover, our confidence in our results relies on the fact that we re-captured the results of van Ingen et al. [16] for some of the isolates analyzed by them, using our unique analysis. For the clinical samples, each of which is clonal, we repeated our main results with the traditional strategy of mapping short reads to reference. Our simulation of mycobacteria mixtures (Appendix A) demonstrated highly accurate strain identification using the same method. We therefore have confidence in our in silico isolation strategy, and believe it can be used in other similar scenarios, in which in vitro isolation is not practical.

Many of the samples taken from HCU water contained *Mycobacterium gordonae*. Interestingly, other groups also found *M. gordonae* in cultures taken from HCU devices [18]. The culture medium used to grow *Mycobacterium* favors the growth of both species, and if the water contained more *M. gordonae* than *M. chimaera*, this would explain why it constituted the majority of sequences in some of our samples.

The global outbreak strain was found in some of the LivaNova/Sorin T3 HCUs in Israel but not in patients. As far as we know, no case of *M. chimaera* infection was diagnosed in a patient who had undergone cardio-thoracic surgery in Israel. This fortunate lack of infected patients, despite the presence of the outbreak strain in local HCU devices, may be due to several factors. The location and orientation of the HCU has a major effect on the chance of infection. If the infected devices were placed outside the operation room, or even inside it, but in an orientation such that the airflow direction is away from the surgery bed, this could diminish the infection risk [14]. Even in medical centers where cardio-thoracic surgery caused infections, these infections were very rare [26,27]. Lastly, several years can pass after the surgery before the infection manifests clinically [11,15].

Studies similar to ours were conducted globally (e.g., [12,17,18,19]). Our findings are in agreement with other studies, confirming the single strain of *M. chimaera* related to the outbreak and its common source.

Our study included nine *Mycobacterium intracellulare subsp. chimaera* clinical isolates from lungs (sputum or thoracic biopsy). Lung NTM infections are increasing worldwide [28,29,30]. In a previous study, 28% of Mycobacterium avium complex infections identified in human pulmonary samples were caused by *Mycobacterium intracellulare subsp. chimaera* [30]. The importance of this pathogen to human health is, therefore, beyond the cardio-thoracic surgery related outbreak, mostly regarding lung infection (for example [31,32,33]). Our knowledge of the population structure, mode of infection and virulence mechanism of this emerging pathogen is still lacking.

## 4. Materials and Methods

### 4.1. Bacterial Sampling

Sputum and chest biopsy samples were decontaminated for 30 min at room temperature with a 1:1 volume of 4% NaOH or 1:4 volume of 4% H_2_SO_4_, respectively. DDW was added to stop the process, and the samples were centrifuged at 3000× *g* for 20 min and re-suspended in 5 mL of the supernatant. 0.5 mL of the processed sample was inoculated on solid Löwenstein–Jensen (LJ) medium and incubated at 30 °C until observation of growth (up to 8 weeks).

*Mycobacterium* sampling from water was executed according to Public Health England guidelines [34]. One liter from each water sample was centrifuged at 3000× *g* for 20 min, the supernatant discarded and about 4 mL of the remaining was decontaminated using 1:4 volume of 4% H_2_SO_4_ with gentle agitation for 15 min. Decontamination stop, inoculation and incubation of the water samples were identical to the treatment of the clinical samples. Acid fast staining confirmed positive cultures [35].

### 4.2. DNA Extraction and Species Identification by Hain Lifescience Assay Kit

Crude DNA was extracted from the positive cultures by suspending a loop-full of bacteria from LJ medium in 300 µL water and heat inactivation for 45 min at 95 °C, followed by 15 min of sonication in an ultrasonic bath and centrifuging at 13,000 rpm for 5 min. A 5-µL aliquot of the supernatant was used for molecular identification using the GenoType NTM-DR assay (Hain Lifescience, Nehren, Germany) that was performed according to the manufacturer’s instructions.

### 4.3. Genomic DNA Isolation

Two different protocols were used for the isolation of genomic DNA. Manual RFLP-grade DNA extraction was performed, as previously described [36].

An automated DNA extraction was performed by suspending a confluent portion of bacteria from LJ media in 400 µL TE, heat inactivated for 30 min at 90 ℃ followed by incubation with 1 mg/mL lysozyme at 37 ℃ overnight. An amount of 400 µL of the bacterial lysate was transferred to the automated MagNA Pure Compact system for DNA extraction according to the manufacturer’s instructions (Roche Life Science, Penzberg, Germany).

### 4.4. Whole Genome Sequencing

Whole genome sequencing was performed at Hylabs LTD, Israel. Libraries were prepared using the NEB Ultra DNA library prep kit. Ten of the samples were sequenced on Illumina HiSeq instrument and 13 of the samples were sequenced on Illumina MiSeq instrument. Both sequencing generated 2 × 150 bp paired end reads.

### 4.5. Metagenomic Taxon Annotation of Reads

Annotating each short-read sequence to a taxon was undertaken with Kaiju [37]. In essence, each read was compared to a database of publically available DNA sequences to assign a taxon to it. When a high-resolution taxon level assignment was not possible, a lower resolution level was used. (For example, when it could not be decided to which species a read belongs, Kaiju would try to decipher to which genus it belongs. If a genus level annotation could not be made, a family level annotation would be tried, and so on).

### 4.6. Metagenomic Binning

Short reads were de novo assembled into contigs using SPAdes v3.11.1 [38]. The Assemblies were uploaded to the Pathosystems Resource Integration Center (PATRIC, https://www.patricbrc.org/; https://www.patricbrc.org/, accessed on 24 January 2019) [39], and PATRIC ‘Metagenomic Binning’ service was applied. The metagenomic algorithm used by PATRIC is explained in [38]. Briefly, a data base of representing sequences of a protein, encoded by a single gene in all prokaryotic genomes, is searched against for initial identification of the species present in the sample (the ‘bins’). Each bin has a reference genome associated with it. The assembly contigs are then assigned to the bins by Blast against these reference genomes.

To identify the species of each retrieved assembly bin, we used PATRIC service ‘Similar Genome Finder’, which implements the Mash tool [24]. The Mash algorithm represents each genome by a sketch, containing 1000 k-mers from this genome. The higher the similarity between two genomes, the more k-mers their sketches have in common.

### 4.7. SNP Calling and Genotype Calling

The *dnadiff* command in the MUMmer suit [40] was used to align each genome assembly to the genome assembly of *M. chimaera* reference strain DSM-44623 (RefSeq accession NZ_CP015278.1), and find variant positions. For each sample, the output of this command was a list of variant positions, relative to the reference. SNPs were filtered if another SNP existed within a window of 12 bp in the same genome. We constructed a dataset of all the SNPs identified from all the isolates in this study, and from a selection of 40 isolates analyzed by van Ingen et al. [16]. The database contains a total of 67,762 SNPs.

Since *dnadiff* only reports variant positions, it did not enable us to distinguish between cases where the sample genome is identical to the reference at a certain locus, and cases where this locus is not covered in the sample genome.

**Figure 2:**
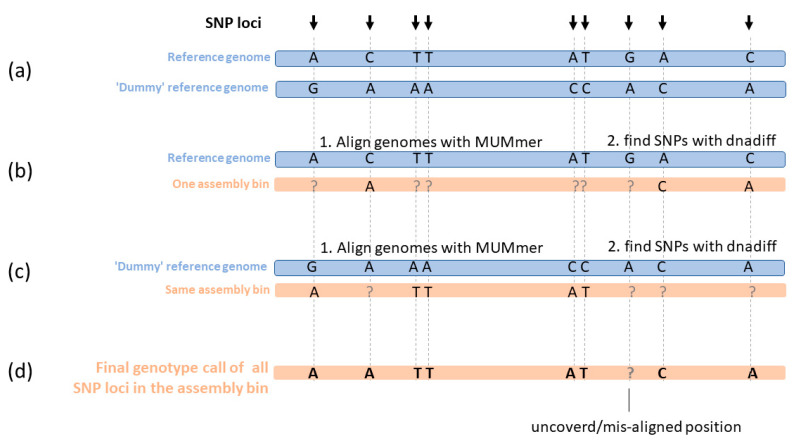
The process of genotype call. The assembled genome from each sample was aligned against the reference genome and SNPs were called. The SNPs from all the samples were used to build one dataset of SNPs. In order to differentiate SNP loci where the isolate genome is identical to the reference genome, from loci not covered or not properly aligned in the isolate, each isolate was also compared to the ‘dummy’ reference genome. (**a**) The ‘dummy’ reference genome was created by changing the base in each of the dataset loci. (**b**) Positions from the dataset in which the isolate is different from the reference genome were identified when aligning the isolate genome to the true reference genomes and the isolate base call in these positions was recorded. Positions with a question mark are those where the isolate is either identical to the reference genome, or the two genomes did not align. (**c**) The alignment step was repeated using the ‘dummy’ reference genome. (**d**) The genotype calls from (**b**) and (**c**) are integrated so a final genotype call is given to all SNPs in the dataset where the isolate genome aligns with the reference genome.

To distinguish between these two cases, we applied the following procedure (Figure 2):

A. We created a ‘dummy’ reference genome, in which the original bases in the reference genome of strain DSM-44623 were changed in all SNP loci, to some other base. All other loci in the ‘dummy’ reference genome are identical to the original reference genome. There is no significance to the base chosen to replace the reference base, as long as it is not the same (Figure 2a).

Genotype calls were collected as follows:

B. As mentioned above, for each isolate, it assembly bin was aligned to the reference genome, and SNPs were identified using *dnadiff* command in the MUMmer suit. For each SNP in the dataset, if the isolate differs from the reference genome in this base position, this base position appears in the output with the genotype call of the isolate.

C. Similar to the previous step, each isolate was aligned to the ‘dummy’ reference genome, and SNPs were identified. Since the ‘dummy’ genome differs from the true reference genome in all the SNPs in the dataset, for each SNP in the dataset where the isolate base is identical to the true reference genome, it differs from the ‘dummy’ reference genome and this base call appears in the output of *dnadiff* (Figure 2c).

D. Genotype call for each isolate from all dataset SNP loci was integrated from both B and C (Figure 2d).

### 4.8. SNP Based Phylogenetic Tree

After merging isolates of identical genotype, the SNPs were concatenated into a DNA sequence. A maximum likelihood phylogenetic tree was calculated using RAxML [41] with General Time Reversible model of nucleotide substitution under the Gamma model of rate heterogeneity with ascertainment bias correction, followed by 100 bootstrap iterations.

### 4.9. Group Specific SNP Signatures

The researchers van Ingen et al. identified SNPs signatures specific to some of their genotype groups and sub-groups and included them in Table S2 in their paper [16]. We searched our genomes for these signatures. We compared the bases in Table S2 from van Ingen et al. to the genome sequences strains DSM-44623 and ZUERICH-1 (RefSeq accessions NZ_CP015278.1 and NZ_CP015272.1, respectively). We noticed a mistake in one of the SNPs in the table, the reference allele in position 4,050,336 is C, not G, and the table was corrected accordingly. SNP genotypes were called, as described above. Isolates were identified which contain group-specific SNP signatures.

### 4.10. Group Specific Signature SNP in Clinical Samples, Using Mapping of Reads to Referecne Genome

In order to validate our results, we repeated the SNP identification in our clinical samples only, in a more mainstream approach. Reads were aligned to DSM-44623 reference genome with botwie2 [42], Samtools [43] and Varscan [44] were used for SNP identification. The genotype in each signature SNP loci was compared to the one obtained from metagenomic binning strategy.

## 5. Conclusions

The global outbreak strain was found in some of the LivaNova/Sorin T3 HCUs in Israel but not in patients. The use of metagenomic binning enabled strain identification from mixed cultures. The characterization of clinical *M. chimaera* isolates in Israel is important for our ability to surveil this emerging pathogen.

## Figures and Tables

**Table 1:** A list of the samples sequenced and analyzed in this study.

Sample Number	Source ^1^	DNA Extraction ^2^	Illumina Sequencing Instrument ^3^	PCR Based Identification ^4^	Year of Sampling	HCU Production Date ^5^	HCU Number	Medical Center ^6^
M1	cl.: sputum	RFLP	HiSeq	*M. chimaera*	2017			Tm
M2	cl.: sputum	RFLP	HiSeq	*M. chimaera*	2017			Ma
M3	cl.: sputum	RFLP	HiSeq	*M. chimaera*	2017			Abct
M4	cl.: sputum	Magnapure	MiSeq	*M. chimaera*	2017			Tlm
M5	cl.: sputum	Magnapure	MiSeq	*M. chimaera*	2017			Ma
M6	cl.: sputum	Magnapure	MiSeq	*M. chimaera*	2017			Nt
M7	cl.: sputum	RFLP	MiSeq	*M. chimaera*	2017			Rm
M8	cl.: sputum	RFLP	MiSeq	*M. chimaera*	2017			Nt
M9	cl.: thoracic biopsy	RFLP	MiSeq	*M. chimaera*	2014			-
M10	cl.: pleural fluid	RFLP	HiSeq	*M. intracellulare*	2013			-
M11	HCU	Magnapure	MiSeq	*M. chimaera*	2017	2011	16S12916	Lc
M12	HCU	Magnapure	MiSeq	*M. chimaera*	2017	2004	16S10462	Lc
M13	HCU	Magnapure	MiSeq	*M. chimaera*	2017		16S12007	Nk
M14	HCU	Magnapure	MiSeq	*M. chimaera*	2017	2004	16S10468	Ahs
M15	HCU	Magnapure	MiSeq	*M. chimaera*	2017	2011	16S12918	Rs
M16	HCU	RFLP	HiSeq	*M. chimaera*	2017	2007	16S10888	Lc
M17	HCU	RFLP	HiSeq	*M. chimaera*	2017	2004	16S10448	Nw
M18	HCU	RFLP	HiSeq	*M. chimaera*	2017	2007	16S10890	As
M19	HCU	RFLP	HiSeq	*M. chimaera*	2017	2004	16S10395	Hh
M20	HCU	RFLP	HiSeq	*M. chimaera*	2017		16S10082	Ks
M21	HCU	RFLP	HiSeq	*M. chimaera*	2017	2014	16S14090	Aa
M22	HCU	Magnapure	MiSeq	*M. chimaera + M. gordonae*	2017	2015	16S15449	Ap
M23	HCU	Magnapure	MiSeq	*M. chimaera + NTM*	2017	2015	16S15447	Nw

^1^ Source: cl. = clinical. ^2^ The genomic DNA was extracted using two methods (‘RFLP’ and ‘Magnapure’, see methods). ^3^ Sequencing was performed using Illumina short reads technology in either a HiSeq or a MiSeq instrument (see methods). ^4^ PCR based identification-species identification by Hain Lifescience GenoType NTM-DR assay. ^5^ The production date of the HCU device, when available. ^6^ The hospital or the clinic in which the HCU was used, or the clinical sample was obtained, when available.

**Table 2:** Species identification.

Sample Number	Source ^1^	PCR Based Identification ^2^*	Reads’ Most Abundant Species ^3^*	Reads’ *M. chimaera* Abundance ^4^*	Number of Bins ^5^	Most Similar Genome to Bin (# K-mers) ^6^*
M1	cl.: sputum	*M. chimaera*	*M. chimaera* (3.6%)	3.6%	1	*M. chimaera* strain MCIMRL2 (920)
M2	cl.: sputum	*M. chimaera*	*M. chimaera* (0.8%)	0.8%	1	*M.* sp. TKK-01-0059 (874)
M3	cl.: sputum	*M. chimaera*	*M. chimaera* (6.4%)	6.4%	1	*M. chimaera* strain ZUERICH-2 (896)
M4	cl.: sputum	*M. chimaera*	*M. chimaera* (4.4%)	4.4%	1	*M. chimaera* strain ZUERICH-1 (805)
M5	cl.: sputum	*M. chimaera*	*M. chimaera* (3.8%)	3.8%	1	*M. chimaera* strain ZUERICH-1 (325)
M6	cl.: sputum	*M. chimaera*	*M. chimaera* (4.6%)	4.6%	1	*M. chimaera* strain DSM 44,623 (880)
M7	cl.: sputum	*M. chimaera*	*M. chimaera* (4.4%)	4.4%	1	*M. chimaera* strain DSM 44,623 (893)
M8	cl.: sputum	*M. chimaera*	*Shewanella decolorationis* (5.6%)	4.2%	1	*M. chimaera* strain DSM 44,623 (903)
M9	cl.: thoracic biopsy	*M. chimaera*	*Bacillus azotoformans* (6.6%)	3.9%	1	*M. chimaera* strain DSM 44,623 (873)
M10	cl.: pleural fluid	*M. intracellulare*	*M. intracellulare* (6.2%)	0.2%	1	*M. intracellulare* MIN_061107_1834 (801)
M11	HCU	*M. chimaera*	*M. chimaera* (3.4%)	3.4%	1	*M. chimaera* strain CDC 2015-22-71 (987)
M12	HCU	*M. chimaera*	*M. chimaera* (3.6%)	3.6%	1	*M. chimaera* strain CDC 2015-22-71 (987)
M13	HCU	*M. chimaera*	*M. chimaera* (10.3%)	10.3%	1	*M. chimaera* strain ZUERICH-2 (963)
M14	HCU	*M. chimaera*	*M. chimaera* (4.7%)	4.7%	1	*M. chimaera* strain ZUERICH-1 (1000)
M15	HCU	*M. chimaera*	*M. chimaera* (4.1%)	4.1%	1	*M. chimaera* strain ZUERICH-1 (999)
M16	HCU	*M. chimaera*	*M. gordonae* (15.1%)	0.6%	2	*M. gordonae* strain 1275229.4 (567)
						*M. chimaera* strain ZUERICH-1 (900)
M17	HCU	*M. chimaera*	*M. gordonae* (18.5%)	0.2%	2	*M. gordonae* strain 1275229.4 (609)
						*M. chimaera* strain WCHMC000032 (841)
M18	HCU	*M. chimaera*	*M. gordonae* (17.7%)	0.3%	2	*M. gordonae* strain 1275229.4 (609)
						*M. chimaera* strain WCHMC000032 (841)
M19	HCU	*M. chimaera*	*M. gordonae* (12.5%)	0.2%	2	*M. gordonae* strain 1275229.4 (352)
						*M. chimaera* strain SJ42 (743)
M20	HCU	*M. chimaera*	*M.* sp. (6.8%)	0.9%	2	*M.* sp. strain DS2.013 (767)
						*M. chimaera* strain WCHMC000030 (897)
M21	HCU	*M. chimaera*	*M. gordonae* (17%)	0.3%	2	*M. chimaera* strain WCHMC000032 (867)
						*M. gordonae* strain 1275229.4 (645)
M22	HCU	*M. chimaera + M. gordonae*	*M. gordonae* (18.6%)	0.9%	1	*M. gordonae* strain 1275229.4 (605)
M23	HCU	*M. chimaera + NTM*	*M. gordonae* (16.6%)	0.3%	1	*M. gordonae* strain 1275229.4 (562)

^1^ Sample Source: cl. = clinical. ^2^ Species identification by Hain Lifescience GenoType NTM-DR assay. ^3^ The most abundant annotation of short reads on the species level (proportion of reads annotated to this species). ^4^ proportion of reads annotated as M. chimaera.^5^ Number of assembly bins retrieved from metagenomic binning. ^6^ The most similar genome to each bin, as identified by running MASH [24] against a database of all publicly available genomes. MASH uses a sketch in the form of 1000 K-mers to represent each genome. The more K-mers two genomes have in common- the more similar they are. # (Number of common K-mers out of maximum 1000 K-mers). * M. = Mycobacterium.

## Data Availability

The data presented in this study are openly available in ENA at doi:10.15468/avkgwm, project accession number: PRJEB39994.

## Supplementary Materials

The following are available online at https://www.mdpi.com/article/10.3390/pathogens10111392/s1. Table S1: The metagenomic bins and their quality [39]. Table S2: Metadata of M. chimaera samples used for in silico mix simulation. Table S3: Metadata of M. gordonae samples used in the in silico mix simulation. Table S4: Bins constructed from the in silico mixtures of reads from both Mycobacterium chimaera and Mycobacterium gordonae. Table S5: The metagenomic bins from the in silico mixture samples and their quality. Table S6: SNP Genotype call identity between M. chimaera bins from the read mixtures, and bins from the corresponding non-mixed *M*. *chimaera* sample. Table S7: SNP Genotype call identity between M. chimaera bins from the read mixtures and mapping non-mixed reads from the same sample to reference genome. Figure S1: SNP based phylogenetic tree with bins from mixed samples and the non-mixed mapped samples. Figure S2: Genotype call identity between bins from mixed samples and the non-mixed mapped strain. Tables S2–S7 and Figure S1 and S2: Results of the simulation [16,42,45].

## Appendix A

Validation of SNP genotype call and strain identification from mixtures using metagenomic binning.

Since our Environmental samples contained mixtures of *Mycobacterium intracellulare subsp. chimaera* (*M. chimaera*) and *Mycobacterium gordonae (M. gordonae)*, we have developed an in-house method to separate sequence data of mixed samples and identify the strain of *M. chimaera*. Our method includes de novo assembly of reads and metagenomic binning of contigs into different species, followed by genome alignment for SNP detection and genotype call (see methods section in the manuscript). In order to identify this method’s strength and limitations, we applied it to simulated in silico mixed samples with pre-defined proportions of *M. chimaera* and *M. gordonae.*

Short reads from 14 genetically diverse *M. chimaera* samples from van Ingen et al., [16] (Table S2) and 3 different *M. gordonae* from a Mycobacteria species sequencing project (https://www.ncbi.nlm.nih.gov/bioproject/PRJNA401515, accessed on 17 September 2021 Table S3) were used to build 126 mixtures. Each pair of *M. chimaera* and *M. gordonae* constructed three mixtures, with 1:1, 3:1 and 9:1 proportions of *chimaera*:*gordonae*, respectively.

Each mixture was de novo assembled and subjected to metagenomic binning, as described in the method section. The PATRIC metagenomic binning service (see methods) constructed one *M*. *gordonae* bin in each of the 1:1 mixtures. In some of them, a *M. chimaera* bin was also constructed (Tables S4 and S5). In almost all 3:1 mixtures, one *M. chimaera* bin was constructed. In a few, a *M. gordonae* bin was also constructed. In a few, no bin was constructed. In almost all 9:1 mixtures, one *M. chimaera* bin was constructed. A *M. gordonae* bin was constructed in none of them. In a few mixtures, no bin was constructed. No bin annotated other than *M. chimaera* or *M. gordonae* was constructed in any mix (Tables S4 and S5). The ability of the Metagenomic binning service to identify and construct bins is, therefore, influenced by the amount of sequence from each species, as well as characteristic of the sample itself, either its quality or the strain of the species, or a combination of these factors. This explains why a more sensitive PCR-based method (Hain Lifescience GenoType NTM-DR assay) was able to identify two different *Mycobacterium* species in samples M22 and M23 in the main manuscript, whereas metagenomic binning was only able to retrieve an *M. gordonae* bin. However, as shown below, whenever a *M. chimaera* bin was constructed, it was possible to correctly identify its strain.

Subsequently, we continued to evaluate how well our pipeline performs in identifying the strain of the *M. chimaera* bins.

All bins annotated as *M. chimaera* originating from mixtures were used for SNP call and genotype call, as described in the manuscript method section. A total of 30242 SNPs were identified, relative to the reference genome of strain DSM-44623.

In our manuscript, we assigned some *M. chimaera* isolates, obtained from HCU water in Israel, to the outbreak strain. We based the assignment on their position in the phylogenetic tree, which was among samples previously shown by van Ingen et al. [16] to belong to this outbreak strain. We, therefore, wanted to validate that *M. chimaera* bins obtained from in silico mixtures indeed group together on a phylogenetic tree, according to the strain present in the mixture. Figure S1 shows a phylogenetic tree of the *M. chimaera* bins from the mixtures as well as non-mixed samples, color coded by the identity of the original strain in the mixture. Note that strains indeed cluster together correctly. Moreover, strains 16 and 31, and strains 206 and 207, which clustered together in this phylogenetic tree, also cluster together in the phylogenetic tree constructed by van Ingen et al. in their original paper [16]. The phylogenetic analysis, thus, supports our confidence in the method we have chosen.

Among the 30242 SNPs identified in the simulation, the genotype call of each bin was compared to that of its corresponding non-mixed *M. chimaera* strain. The rates of genotype call identity are presented in Table S6. The average genotype call identity between a bin from a mixed sample, and a bin from the clean sample of the same strain, is 99.7%. In comparison, the average identity between a bin from a mixed sample and a bin from a different strain is only 75.0%. Since strain identification is based on SNP genotype calls, this strongly supports the validity of this method for strain identification.

We also wanted to compare the genotype calls obtained from mixtures going through the binning process to those obtained by a more conventional method, namely, mapping the short sequence reads from non-mixed sample to the reference genome. The short reads from the 14 *M. chimaera* samples used in the simulation were re-mapped to the DSM-44623 reference genome with botwie2 [42]. Samtools and BCFtools [45] were used for per-position genotype call. Genotype calls were filtered for cover higher than 4, and ratio higher than 0.9 between major allele likelihood and minor allele likelihood. Genotype calls in the loci of SNPs identified among bins were obtained. We compared the genotype calls from the mixtures undergoing assembly and metagenomic binning, to the genotype calls obtained from their respective non-mixed samples, undergoing re-mapping (Table S7, Figure S2). On average, the genotype call identity was 98%. In comparison, the genotype call identity between a mixture-originating bin and a non-mixed re-mapped sample of a different strain is on average 73% (Table S7), again supporting the validity of strain identification using our method.

In summary, the validation demonstrates that our method is capable of separating different strains, and correctly identifying samples belonging to the same strain, thus assuring the validity of our results.

Our method is, however, not without limitations. The main limitation is the failure to identify bins of all species present in some mixtures.
