# Supplementary material for: Mycobacterium intracellulare subsp. chimaera from Cardio Surgery Heating-Cooling Units and from Clinical Samples in Israel Are Genetically Unrelated
Source: Pathogens. 2021 Oct 27;10(11):1392. doi: 10.3390/pathogens10111392 (PMC8624631; doi:10.3390/pathogens10111392)
Supplement: Supplementary file 1 [file pathogens-10-01392-s001.zip › pathogens-1353724-supplement .pdf]

Supplementary Tables:

Table S1: The metagenomic bins and their quality. Metagenomic binning was done using the PATRIC service with the identical name (Pathosystems Resource Integration Center, https://www.patricbrc.org/) [39]. A detailed explanation of the algorithm used by this service is available in https://docs.patricbrc.org/tutorial/binning_overview.html . Columns 10-18 ('Score' – 'Good PheS') are derived from the summary table in PATRIC for metagenomic binning results. The description of these columns is available in https://docs.patricbrc.org/tutorial/metagenomic_binning/metagenomic_output.html#the-binning-output-directory. The table also contains the GC content of each bin, the number of coding sequences identified (PATRIC CDS), and the number of tRNA and rRNA coding genes found. The last pair of columns indicate whether the bin was included in the phylogenetic analysis and searched for group-specific SNPs, and the sub-type according to these signature SNPs, if found.

Table S2: Metadata of *M. chimaera* samples used for *in silico* mix simulation. Data taken from suppplementary table 1 in van Ingen et al [16].

Table S3: Metadata of *M. gordonae* samples used in the *in silico* mix simulation. Data taken from SRA database (https://www.ncbi.nlm.nih.gov/sra), from which short sequences were obtained.

Table S4: Bins constructed from the *in silico* mixtures of reads from both *Mycobacterium chimaera* and *Mycobacterium gordonae*. The number of *M. chimaera* bins, *M. gordonae* bins and other species bins constructed for each mixture.

Table S5: The metagenomic bins from the *in silico* mixture samples and their quality. Metagenomic binning was performed using the PATRIC service. Details are the same as Table S1.

Table S6: SNP Genotype call identity between *M. chimaera* bins from the read mixtures, and bins from the corresponding non-mixed *M. chimaera* sample. SNPs were identified by comparing all the mix simulation bins to the reference genome and bin's genotype calls were determined, as described in the manuscript method section. SNP Genotype call from bins constructed from the in silico simulated mixtures were compared to the genotype call of bins from the same, non-mixed strain. Cells show the rate of SNP call identity.

Table S7: SNP Genotype call identity between *M. chimaera* bins from the read mixtures and mapping non-mixed reads from the same sample to reference genome. SNPs were identified by comparing all the mix simulation bins to the reference genome and bin genotype calls were determined as described in the manuscript method section. Non-mixed samples were mapped to reference genome and genotype calls for the same loci were obtained, as described in the appendix. For each bin, its genotype call was compared to the calls from the corresponding non-mixed *M. chimaera* strain mapped reads. Columns 1-4 contain mixture details. The number of identical genotype calls and the number of different genotype calls between the bin from the mixture and the corresponding non-mixed mapped sample are in columns 5 and 6 respectively. Columns 7 contains the number of loci in which the mixture bin had a genotype call, but were not covered or filtered out in the mapped reads. Column 8 has the rate of identical genotype calls, and column 9 has the average identical genotype call when comparing the bin to non-mixed mapped reads from other strains.

Supplementary Figures:
Figure S1: SNP based phylogenetic tree with bins from mixed samples and the non-mixed mapped samples. Sample names are color-coded according to the *M. chimaera* strain present in the mixture. A. all samples. B. Group 1 samples only.

Figure S2: Genotype call identity between bins from mixed samples and the non-mixed mapped strain. A box-plot showing the distribution of genotype call identity between *M. chimaera* bins from the simulated mixtures, and mapping the short reads of the same non-mixed strain directly to the reference genome. The results are shown for the 3 proportions of *M. chimaera:M. gordonae* in the mixtures.
