# Supplementary figures and images for: Mycobacterium intracellulare subsp. chimaera from Cardio Surgery Heating-Cooling Units and from Clinical Samples in Israel Are Genetically Unrelated
Source: Pathogens. 2021 Oct 27;10(11):1392. doi: 10.3390/pathogens10111392 (PMC8624631; doi:10.3390/pathogens10111392)

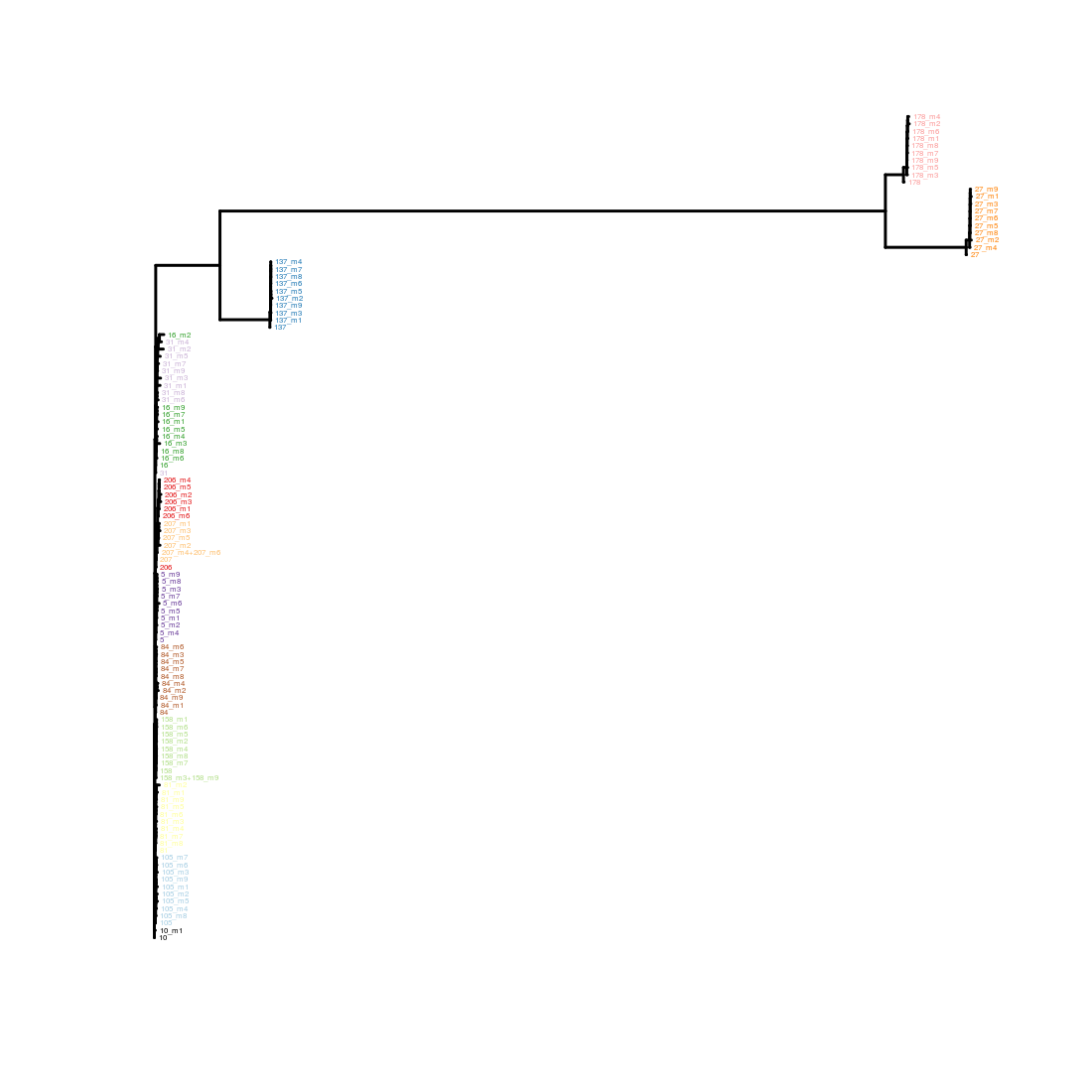

Supplement: Supplementary file 1 [file pathogens-10-01392-s001.zip › supplementary/Figure_S1a.png]

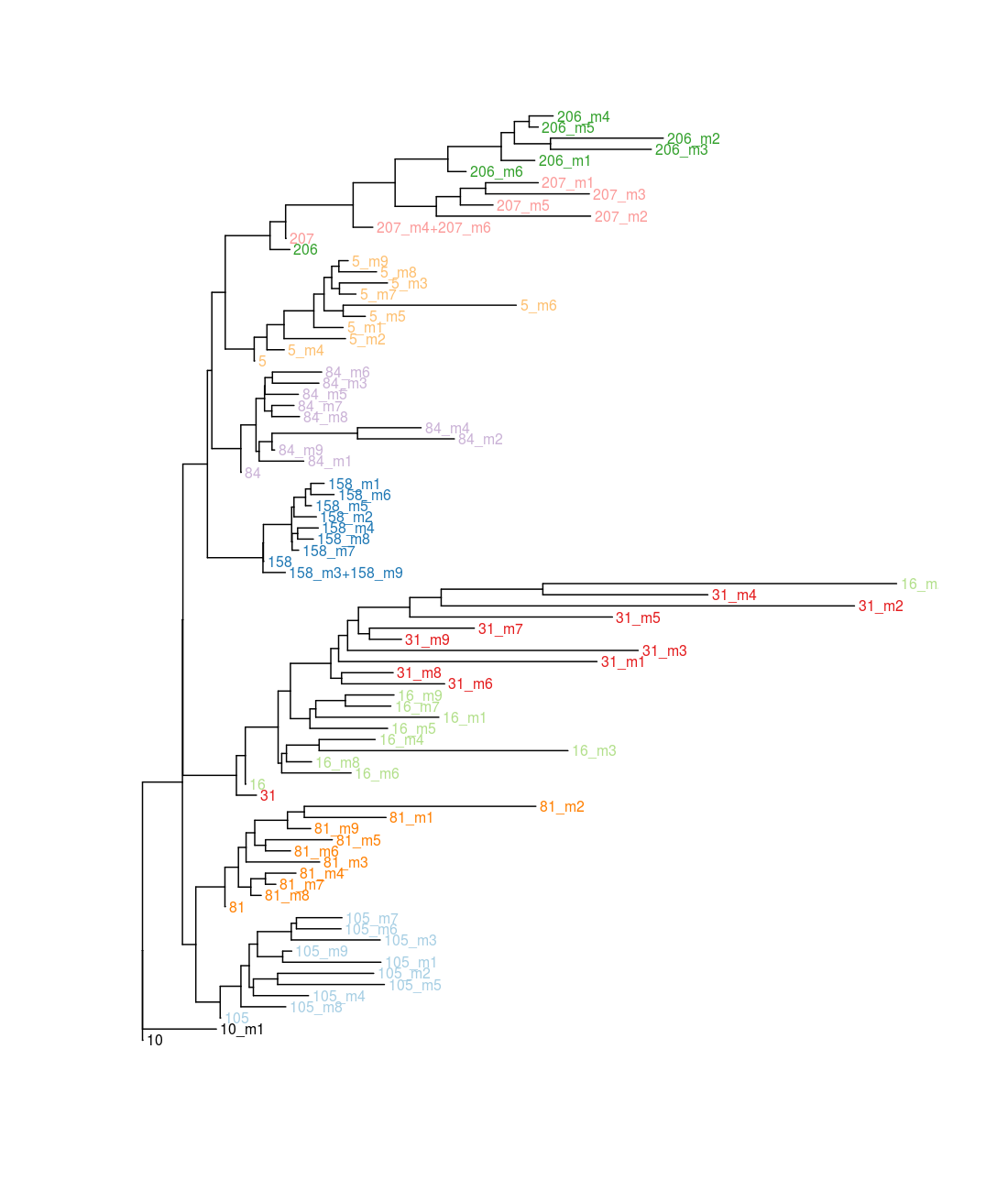

Supplement: Supplementary file 1 [file pathogens-10-01392-s001.zip › supplementary/Figure_S1b.png]

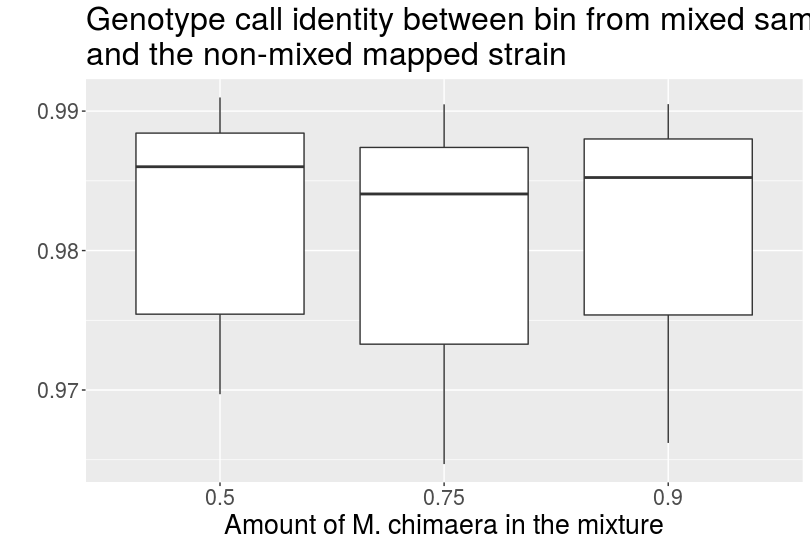

Supplement: Supplementary file 1 [file pathogens-10-01392-s001.zip › supplementary/Figure_S2.png]
